# A Treatise on Food and Dietetics, Physiologically and Therapeutically Considered

**Published:** 1874-08-15

**Authors:** 


*Received through Jansen, McClurg & Co.*

A TREATISE ON FOOD AND DIETETICS, PHYSIOLOGICALLY AND THERAPEUTICALLY CONSIDERED. By F. W. Panz, M.D., F.R.S. Henry C. Lea: Philadelphia.

There has long been an opening and a need for just such a work as the author here places before us—a thorough, complete, scientific treatise on Food and Dietetics in their physiological and therapeutical relations.

In the introductory chapters on the *Dynamic Relations of Food*, the laws governing force and matter are briefly considered; also, the influence of solar force, the action of vegetable life, etc.

The Alimentary Principles: their classification and chemical relations, are next considered.

In reference to Leibig's theory regarding the nitrogenized and non-nitrogenized principles, which classes the former as "plastic elements of nutrition," and the latter as simply designed for undergoing oxidation, and in this way serving as a source of heat, the author says, "although nitrogenized principles constitute true elements of nutrition, yet it neither follows nor appears likely that they are limited to this purpose. Fats are undoubtedly important calorifacient principles, and cannot *per se* supply what is required for tissue development; they, nevertheless, take part in the process.

The relations of exercise to the elimination of nitrogen are also fully and extensively considered. Dr. Austin Flint, Jr., from his experiments and observations during the celebrated walking feats of the pedestrian Weston, came to the conclusion that "excessive and prolonged muscular exertion increased enormously the excretion of nitrogen, and that the excess of nitrogen discharged was due to an increased dis-assimilation of the muscular substance."

The author's views, however, differ from those of Dr. Flint, and an abundance of testimony is adduced, apparently going to show that muscular action should not be considered as the result of muscular destruction, as was formerly supposed, and hence that nitrogenous matter is not applied through muscle to the development of muscular force. While he does not consider, then, that the elimination of urea is influenced by muscular action, it does bear a marked relation to the food ingested. Urea is regarded as constituting the unutilized portion of the nitrogenous principles. "About one-seventh," he says, "of the potential (latent) energy—capacity for force production—belonging to nitrogenous matter, is carried off as urea." The greater the amount of nitrogenous food ingested, therefore, the larger the amount of urea that will be excreted. This principle would seem to be borne out by the facts and experiments as given by the author.

A suggestion here thrown out regarding the uses and mode of formation of fat in the system, is of interest: "Seeing that nitrogenous matter is broken up, 1st, into a nitrogenous portion—urea—which is eliminated as useless, and, 2nd, a hydrocarbonaceous residue which represents capacity for force production, the question next confronts us, whether this hydrocarbonaceous residue, instead of being oxydized and applied at the moment of its production, presents itself under a form (that of fat, for example,) for retention in the system, and for application as necessity demands."

All attempts to settle this question by the artificial or chemical productions of fat from protein compounds have heretofore failed.

The non-nitrogenous alimentary principles are considered under the following headings: The hydrocarbons, or fats; the carbohydrates, starch, sugar, etc., and the vegetable acids and alcohol.

We note nothing of especial interest in the views expressed under these heads, except as regards the statements in relation to the action of alcohol on the system. In summing up, the author says, "from a view of the evidence as it at present stands, it may reasonably be inferred that there is sufficient before us to justify the conclusion that the main portion of the alcohol ingested becomes destroyed within the system, and if this be the case, it may be fairly assumed that the destruction is attended with oxidation and a corresponding liberation of force, unless, indeed, it should undergo metamorphosis into a principle to be temporarily retained, but, nevertheless, ultimately to be applied to force production."

The consideration of these alimentary principles is completed in the first 145 pages of the book. The remainder of the volume, some four hundred pages, is devoted to the alimentary substances, and contains a careful review of the actual and comparative value of all the principle animal and vegetable substances ordinarily used as food. F. H. D.

---

THE CHICAGO JOURNAL OF NERVOUS AND MENTAL DISEASE.

The July number of this journal has been received. Its contents are fully equal in value and importance to that of the two preceding numbers. The third lecture on the Pathology of the Vaso-Motor System by the senior editor, forms the leading article. Three other valuable original articles, with reviews, editorial and a very extended and carefully prepared periscope, form altogether a volume of 142 pages.

---

ZOSTER THORACICALIS—*Treatment by the Faradic Current, by A. D. Rockwell.*—Jane A., a dispensary patient, aged seven years, six months, had suffered for several weeks from febrile symptoms and anorexia, and finally erythematous patches appeared on the chest and right side. The eruption increased and rapidly extended, until the thorax was nearly encircled. The pain from which the child suffered was very severe, and for forty-eight hours it had been continuous. I employed general faradization (mildly) and was rewarded by an immediate relief of the neuralgic pains. Four similar applications were subsequently given,—one on each alternate day; but there was no return of pain, and within ten days the eruption, which resembled aborted vesicles, had quite disappeared.—*Phila. Med. Times.*

